# Asia-Pacific multicentre randomized trial of laparoscopic *versus* open major hepatectomy for hepatocellular carcinoma (AP-LAPO trial)

**DOI:** 10.1093/bjsopen/zrac166

**Published:** 2023-02-28

**Authors:** Kelvin K C Ng, Charing C N Chong, Kit-Fai Lee, Paul B S Lai, Thomas K C Cheng, Hua-Wei Chen, Bin Yi, Ji-Wei Huang

**Affiliations:** Department of Surgery, The Chinese University of Hong Kong, Hong Kong; Department of Surgery, Prince of Wales Hospital, Hong Kong; Department of Surgery, The Chinese University of Hong Kong, Hong Kong; Department of Surgery, Prince of Wales Hospital, Hong Kong; Department of Surgery, Prince of Wales Hospital, Hong Kong; Department of Surgery, The Chinese University of Hong Kong, Hong Kong; Department of Surgery, Prince of Wales Hospital, Hong Kong; Department of Surgery, Kwong Wah Hospital, Hong Kong; Department of Surgery, The First People Hospital of Foshan, Foshan, China; Department of Biliary Surgery, Eastern Hepatobiliary Surgery Hospital & Institute, Second Military Medical University, Shanghai, China; Department of Surgery, West China Medical School of Sichuan University, Sichuan, China

## Abstract

**Background:**

Hepatocellular carcinoma is the sixth most common malignancy in the world. Major hepatectomy (resection of greater than or equal to three liver segments) is needed if a tumour is large or close to major blood vessels. Despite low mortality, open major hepatectomy is associated with high rates of tumour recurrence that limits survival. Laparoscopic major hepatectomy has been proposed as an alternative approach with potential oncological benefits. This study compares laparoscopic major hepatectomy with open major hepatectomy for hepatocellular carcinoma in a randomized trial.

**Methods:**

The Asia-Pacific multicentre randomized trial of laparoscopic *versus* open major hepatectomy for hepatocellular carcinoma (AP-LAPO trial) is an open-labelled multicentre randomized trial to be conducted in five centres in the Asia-Pacific region. The study will test the hypothesis that laparoscopic major hepatectomy for hepatocellular carcinoma is associated with less tumour recurrence and better survival compared with open major hepatectomy; the primary outcome being 2-year recurrence-free survival. Secondary outcomes include hospital mortality, postoperative complications according to the Clavien–Dindo classification, time to functional recovery, quality of life, long-term survival, and postoperative serum surgical stress-related cytokines.

**Results and conclusion:**

The AP-LAPO trial will determine whether laparoscopic major hepatectomy offers oncological benefits to patients with hepatocellular carcinoma compared with open major hepatectomy.

**Registration number:**

NCT04852211 (http://www.clinicaltrials.gov) registered on 21 April 2021

**Protocol version:**

AP-LAPO trial version 01 (1 December 2021)

## Teaser

The AP-LAPO trial will be the first multicentre randomized trial evaluating the long-term oncological benefits of LMH for HCC. It provides level 1 evidence on the best surgical approach of major hepatectomy for HCC.

## Introduction

Hepatocellular carcinoma (HCC) is the sixth most common malignancy in the world^1^. The practice of screening by ultrasonography and serum *α*-fetoprotein concentration in cirrhotic patients and hepatitis B carriers has led to an increasing incidence of HCC^2^. Curative treatment options for patients with early HCC include hepatic resection, local ablation, and liver transplantation. Hepatectomy remains the mainstay treatment for patients with HCC and non-cirrhotic liver according to European Association for the Study of the Liver (EASL), American Association for the Study of Liver Diseases (AASLD), and ESMO guidelines^3–5^.

Open major hepatectomy (OMH) (resection of greater than or equal to three liver segments) is indicated for large tumours (greater than 5 cm) that are centrally located and/or close to intrahepatic major vessels. OMH is associated with significant morbidity (about 30 per cent), though hospital mortality has been reduced to less than 5 per cent^6^. Wound and pulmonary complications are the most common early complications, but intrahepatic tumour recurrence remains a major problem in up to 50 per cent of patients^7^.

Laparoscopic major hepatectomy (LMH) has become a feasible and effective approach for the surgical management of HCC^8^. LMH seems to be associated with less surgical stress and manipulation of the tumour during surgery^9^, without increasing blood loss, morbidity, and mortality^10^. These potential benefits might lead to long-term survival benefit^11^ and better quality of life for patients. LMH and OMH have been compared in retrospective matching analyses^8,12^. The aim of this multicentre RCT is to compare LMH and OMH for HCC. The primary outcome is recurrence-free survival.

## Methods

The Asia-Pacific multicentre randomized trial of laparoscopic *versus* open major hepatectomy for hepatocellular carcinoma (AP-LAPO trial) is an open-labelled randomized trial to be conducted in five Asia-Pacific centres (Department of Surgery, The Chinese University of Hong Kong; Department of Surgery, Kwong Wah Hospital, Hong Kong; Department of Surgery, The First People Hospital of Foshan, China; Department of Biliary Surgery, Eastern Hepatobiliary Surgery Hospital & Institute, Second Military Medical University, China; Department of Surgery, Department of Surgery, West China Medical School of Sichuan University). The trial has been registered at ClinicalTrials.gov of the US National Library of Medicine (NCT04852211). The trial protocol follows the Standard Protocol Items: Recommendations for Interventional Trials (SPIRIT) guideline^13^. The coordinating centre for the trial is the Department of Surgery, The Chinese University of Hong Kong. The trial is free of industrial sponsorship.

### Eligibility criteria

Surgical procedures will be carried out by senior hepatobiliary surgeons. According to the international standard for laparoscopic liver surgery, each surgeon will need to have performed at least 55 resections classified as LMH to be included in the study^14,15^.

Inclusion criteria consist of: a diagnosis of HCC as defined by the EASL^16^; tumour size less than or equal to 8 cm or in the case of multiple nodules in a single lobe the sum of the greatest diameter of all nodules less than or equal to 8 cm^12,17,18^; tumour suitable for both LMH and OMH requiring resection of greater than or equal to three Couinaud’s segments as agreed by the hepatobiliary multidisciplinary team; absence of extrahepatic metastasis or radiological evidence of invasion of portal vein or hepatic vein branches; Child A liver function; indocyanine green retention at 15 min (ICG-15) less than or equal to 15 per cent; adequate future liver remnant based on image-guided volumetry greater than or equal to 35 per cent of estimated standard liver volume; and patient fit for general anaesthesia and major liver resection.

**Fig. 1:**
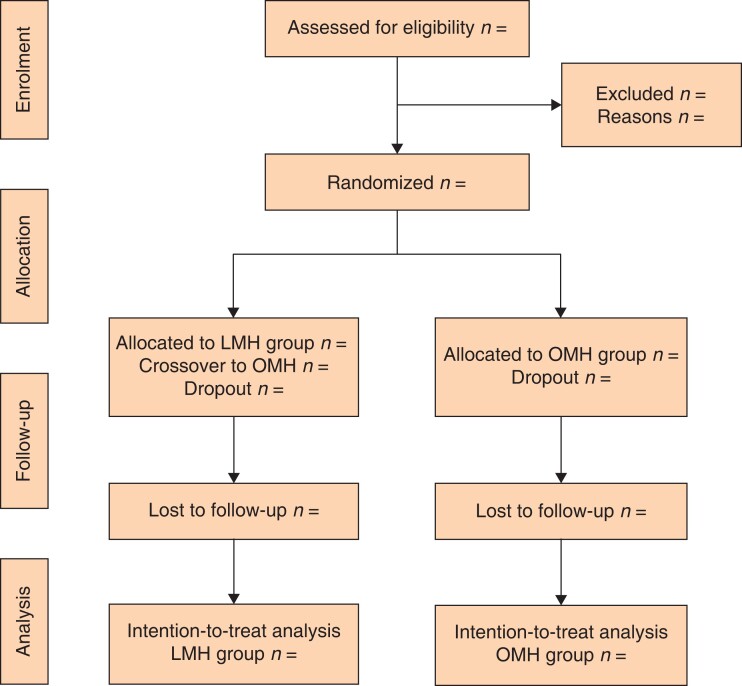
Patient flow chart LMH, laparoscopic major hepatectomy; OMH, open major hepatectomy

Exclusion criteria involve: previous treatment for HCC (for example transarterial chemoembolization or chemotherapy) and tumours requiring combined hepatectomy and thermal ablation therapy. Patients will also be excluded at the time of surgery (dropout) if there is: bilobar liver involvement; unexpected portal vein invasion and peritoneal metastasis not identified at preoperative imaging; liver remnant smaller than expected; or in both laparoscopic and open groups where the operating surgeon decides to abort the procedure due to locally advanced tumour condition before starting the resection phase. The patient flow chart is shown in *Fig. 1*.

### Intervention

Preoperatively all patients will undergo: blood tests (complete blood count, liver and renal function, coagulation profile, serum *α*-fetoprotein level, and hepatitis B and C serology); radiological imaging (chest X-ray, CT scan, or MRI to assess tumour size, number, location, and image-guided volumetry of future liver remnant); ICG-15 test^19^; and quality of life assessment using a Chinese version of the Functional Assessment of Cancer Therapy-General (FACT-G) questionnaires.

All patients will receive prophylactic antibiotics (Augmentin (amoxicillin + clavulanate) (GlaxoSmithKline) 1.2 g intravenously for one dose or levofloxacin (Daiichi Sankyo) 500 mg intravenously for one dose in case of penicillin allergy) and proton pump inhibitor (pantoprazole (Takeda) 20 mg intravenously for one dose). The surgical intervention will be performed under general anaesthesia.

### Interventions: LMH

For the LMH group, the patient will be placed in a left semi-decubitus position. Pneumoperitoneum will be created by carbon dioxide insufflation and the intra-abdominal pressure maintained at 13–15 mmHg. In addition to the camera port (10 mm), two 12-mm and two 5-mm ports will be used and adjusted according to tumour location and extent of resection. Laparoscopic ultrasonography will be used to confirm tumour location and guide liver transection. The extent of resection will depend on tumour location. Cholecystectomy will be performed. The anterior approach for major hepatectomy will be routinely adopted^20^. Following liver hilum dissection to divide the appropriate hepatic artery and portal vein, liver transection will be performed using a combination of ultrasonic dissecting shears (Harmonic scalpel^®^, BBT Medical, Wuhan, China), a vessel sealing system (Ligasure^®^, Medtronic, Minneapolis, MN, USA), radiofrequency energy (TissueLink^®^, Nissha Medical Technologies, Buffalo, NY, USA), and a laparoscopic-adopted Cavitron Ultrasonic Surgical Aspirator (CUSA^®^, Soma Tech, Bloomfield, CT, USA) as determined by the operating surgeon. For vessels greater than 5 mm in diameter, Hem-o-lock or titanium clips will be used. An intermittent Pringle manoeuvre (20 min with 5 min rest for one cycle) will be selectively applied depending on intraoperative haemostasis. The central venous pressure will be routinely kept below 5 mmHg. The hepatic vein and portal pedicles will be transacted by laparoscopic linear stapler. The specimen will be retrieved through a protected wound using Pfannenstiel incision at the suprapubic region. An abdominal drain will be selectively placed on the liver transection surface according to the operating surgeon.

### Intervention: OMH

For the OMH group, resection of the tumour will be performed as previously described^21^. The patient will be placed in a supine position. Laparotomy will be by ‘Mercedes-Benz’ incision. Cholecystectomy will be carried out. Either an anterior or a conventional approach will be adopted according to the preference of the operating surgeon^20^. Hilar dissection to divide the appropriate hepatic artery and portal vein will be performed. Liver transection will be performed using a CUSA^®^ or ultrasonic dissecting shears. Sutures, titanium clips, and vascular staplers will be used to achieve haemostasis. An intermittent Pringle manoeuvre will be selectively adopted. An abdominal drain will be placed according to the operating surgeon.

### Outcomes

#### Primary outcome

The primary outcome is 2-year recurrence-free survival.

#### Secondary outcomes

These include 30-day, 90-day, and in-hospital mortality, postoperative complications, quality of life, time to functional recovery, 2-year overall survival, 5-year overall survival, and recurrence-free survival. Surgical stress-related cytokines serum interleukin 6 (IL-6) and immunosuppressive acidic protein (IAP) will be measured before surgery and on postoperative days 1 and 7. Measurement of IL-6 and IAP levels will be performed by immunoassay using commercially available kits (MILLIPLEX^®^ MAP Human Cytokine/Chemokine Magnetic Bead Panel, Merck Ltd, Rahway, NJ, USA).

### Measurements

Intraoperative: operative time (measured from time of skin incision to wound suturing); blood loss; blood transfusion volumes and other blood products (platelet concentrate, fresh frozen plasma); intraoperative major events (hypotension for greater than 20 min and use of inotropes); and laparoscopic to open conversion. The conversion is defined as the change of surgical approach during the resection phase of major hepatectomy, when the operating surgeon encounters technical difficulty. Patients undergoing conversion will be included in the laparoscopic group and be analysed on an ‘intention-to-treat’ basis.

Postoperative: liver biochemistry and coagulation profile on postoperative days 1, 3, 7, and day of discharge; postoperative morbidities according to Clavien–Dindo classification^22^; 30-day, 90-day, and in-hospital mortality; length of ICU and hospital stay; hospital readmission (90 days after discharge); and time to functional recovery (defined as independently mobile at the preoperative level, sufficient pain control with oral medication alone, ability to maintain at least 50 per cent daily caloric intake, no intravenous fluid administration, and no clinical signs of infection)^23^.

### Follow-up

No adjuvant treatment will be used after surgery. All patients will be followed up every 3–6 months for up to 5 years after operation. Follow-up assessment will include: liver function (clinical assessment of Child’s grade, complete blood count, liver, and coagulation profile); recurrence (based on serum *α*-fetoprotein level, chest X-ray, and CT scan of abdomen with contrast); and quality of life (FACT-G questionnaires).

Patients with intrahepatic recurrence will be managed by laparoscopic re-resection for the LMH group or open re-resection for the OMH group when considered feasible. For patients in whom re-resection is not possible, thermal ablation, transarterial chemoembolization, or salvage liver transplant will be offered according to the decision of the multidisciplinary team.

### Participant timeline

Data accrual will be performed prospectively using wed-based electronic case-report forms (eCRFs) according to the protocol plan (*Table 1* and *Fig. 1*).

**Table 1:** Schedule of enrolment, interventions, and assessments of patients

	Study period					
	Enrolment	Allocation	Post-intervention			Close-out
**Time points**	− 1 month	0	Day 1, 3, 7, Day of discharge	Post-discharge 3, 6, 9, 12, 15, 18, 21, 24 months	Post-discharge 30, 36, 42, 48, 54, 60 months, unscheduled visits	Post-discharge 60 months
**Enrolment**						
Eligibility screening	X					
Informed consent	X					
QoL assessment	X					
Hepatectomy workup	X					
Allocation		X				
**Interventions**						
LMH group			X			
OMH group			X			
Protocol deviation			X			
**Assessments**						
Clinical evaluation including blood tests	X		X	X		
Radiological evaluation	X			X	X	
Postoperative evaluation including mortality and morbidity			X			
Serum *α*-fetoprotein					X	X
Tumour recurrence evaluation					X	X
QoL assessment					X	X
Patient survival evaluation					X	X
Study closure form						X

QoL, quality of life; LMH, laparoscopic major hepatectomy; OMH, open major hepatectomy.

### Sample size

Based on a reported 2-year recurrence-free survival rate of 72 per cent after OMH^12^ and an expected 88 per cent after LMH according to a previously reported series^8^, 150 patients need to be recruited to each arm to demonstrate a statistically significant difference with 80 per cent power at the 0.050 level of two-sided significant difference between LMH and OMH^24^. Allowing a 10 per cent dropout rate, 165 patients will be recruited in each arm. This sample size will allow detection of a reduction of 2-year tumour recurrence from 50 per cent in the OMH group to 30 per cent in the LMH group with a similar power and level of significance. Secondary outcomes will be interpreted for hypothesis-generating only.

### Recruitment

All patients satisfying the inclusion and exclusion criteria will be offered recruitment into the trial. Investigators will provide information on possible advantages and disadvantages of the interventions. Patients will be included only after signing written informed consent (*Appendix S1*). To avoid selection bias, screening log of patients excluded from the trial will be kept.

### Randomization

Preoperative randomization will be adopted on the day of admission. A total of 330 subjects will be allocated 1:1 to either the LMH group or the OMH group. The randomization schedule will be generated by the Clinical Trials Centre (CTC) of the principal investigator’s centre, prior to the start of the study. Stratified block randomization (block size of 10) will be used to ensure treatment allocation in equal proportions at each centre. The randomization list will be kept in a set of tamper-evident envelopes. The envelopes will be identical and sealed. The trial identifier with a sequential number will be printed on each envelope. Envelopes will be unsealed to reveal the allocation treatment 1 day prior to surgery.

### Blinding

There will be no blinding for all recruited patients, investigators, operating surgeons, and other supporting staff.

### Statistical plan

The 2-year recurrence-free survival (primary outcome) and overall survival will be evaluated by Kaplan–Meier method and compared by the log rank test. Other secondary outcomes will be compared using the chi-squared test or Fisher’s exact test for categorical variables, and the Mann–Whitney *U* test for continuous variables. *P* < 0.050 will be considered statistically significant. Statistical analysis will be performed using SPSS version 11.0 (SPSS Inc., Chicago, IL, USA).

All clinical data including patients’ demographics, operative details, tumour characteristics, and clinical outcome measure will be collected and centralized at the principal investigator’s centre using a password-protected web-based system to ensure confidentiality of personal information. Continuous clinical data monitoring will be performed by the principal investigator and co-investigators with the help of biostatisticians and research assistants. The data set will be kept confidential until the publication of trial results.

### Interim analyses

Patient recruitment status will be reviewed every 4 months in planned investigator meetings in which interim analyses will be carried out. If the accrual rate is slower than expected, the principal investigator and co-investigators will discuss the possible reasons and ways to improve the recruitment rate. The interim analyses will not be affected by the results. A data-monitoring group will consist of two independent, external experts who will be responsible for monitoring the progress of the study. If there are significantly more serious adverse events associated with either group after 1 year of commencement of the study, the trial will be terminated as decided by the principal investigator and the data-monitoring group. The same will be applied to the situation where the difference in tumour recurrence between groups is significantly less than expected. The final analysis of the trial will be carried out on an intention-to-treat basis.

### Ethics

An information sheet will be provided, and each subject will be given the opportunity to seek medical advice or to discuss the study with friends or family prior to involvement. Written informed consent will be mandatory for inclusion and patients will be free to withdraw it at any time. This study will be performed in accordance with the ethical standards of the Declaration of Helsinki of 1975 and its later versions. Ethics approval from the institutional review broad of each centre will be obtained.

### Dissemination plan

The protocol of this trial has been registered in Clinicaltrials.gov. The final report on primary and secondary outcomes will be published upon the completion of 5-year follow-up. Both interim analysis and final analysis will be presented at local and international conferences.

## Discussion

The aim of the AP-LAPO trial is to test the hypothesis that LMH is associated with reduced surgical stress and immunosuppression compared with OMH resulting in lower tumour recurrence rates and better survival.

OMH for HCC is associated with major morbidity of up to 40 per cent in high-volume centres^25–28^ as well as a high tumour recurrence rate. The 3-year recurrence rate has been reported to be greater than 50 per cent^7,29^ with most of these recurrences occurring in the liver remnant. Manipulation of the tumour during OMH along with postoperative immunosuppression induced by surgical stress have been suggested as factors that may increase risks of recurrence in the liver remnant^30^.

Recent series have proven the feasibility and safety of LMH for HCC^17^. The conversion and morbidity rates are around 10 per cent. Fewer wound and chest complications and shorter hospital stays are seen after LMH compared with OMH^10^. An animal study has shown that LMH results in less surgical stress and immunosuppression than OMH^9^. Theoretically, laparoscopic instrumentation causes less manipulation of the tumour that might limit cancer-cell dissemination, reducing tumour recurrence and prolonging survival. The reduced immunosuppression might also influence recurrence rates and survival in LMH. This theory is supported by a recent meta-analysis based on reconstructed time-to-event data^11^.

To date, only retrospective studies using propensity score matching have compared LMH and OMH^8,12^. Sample sizes were small (less than 40) in each matched group and the reported 2-year overall and recurrence-free survival were comparable between OMH and LMH. This trial aims to provide high-level evidence to see if there is added value of laparoscopic surgery in the treatment of patients with HCC.

## Trial status

This trial received approval from the Joint Chinese University of Hong Kong—New Territories East Cluster Clinical Research Ethics Committee on 2 September 2020. The trial was registered at ClinicalTrials.gov of the US National Library of Medicine (registration no. NCT04852211) on 21 April 2021. The recruitment began in December 2021 and is anticipated to complete in November 2024.

## Supplementary Material

zrac166_Supplementary_Data

## Data Availability

The data set will be kept confidential until the publication of trial results in an international journal. After that, the data set will be open upon an individual party’s request.
