# Supplementary material for: Asia-Pacific multicentre randomized trial of laparoscopic versus open major hepatectomy for hepatocellular carcinoma (AP-LAPO trial)
Source: BJS Open. 2023 Feb 28;7(1):zrac166. doi: 10.1093/bjsopen/zrac166 (PMC9970867; doi:10.1093/bjsopen/zrac166)
Supplement: zrac166_Supplementary_Data [file zrac166_supplementary_data.docx]

**Asia-Pacific multicentre randomized trial of laparoscopic versus open major hepatectomy for hepatocellular carcinoma (AP-LAPO Trial)**

Kelvin K. C. Ng, *MBBS, MS, PhD, FRCSEd (Gen)*^1,2^ ; Charing C.N. Chong, *MBChB, FRCSEd (Gen)^1,2^* ; Kit-Fai Lee, *MBBS, FRCSEd (Gen)*^2^ ; Paul P. S. Lai, *MBChB, MD, FRCSEd (Gen), FRCS (Glas)*^1,2^; Thomas K.C. Cheng, *MBBS, FRCSEd (Gen)^3^*; Hua-Wei Chen, *MD^4^*; Bin Yi, *MS, PhD^5^*; Ji-Wei Huang, *MD^6^*

Department of Surgery, The Chinese University of Hong Kong, Hong Kong^1^

Department of Surgery, Prince of Wales Hospital, New Territories, Hong Kong^2^

Department of Surgery, Kwong Wah Hospital, Hong Kong^3^

Department of Surgery, The First People Hospital of Foshan, China^4^

Department of Biliary Surgery, Eastern Hepatobiliary Surgery Hospital & Institute, Second Military Medical University, China^5^

Department of Surgery, Department of Surgery, West China Medical School of Sichuan University^6^

**Correspondence and Reprint Request:**

Prof. Kelvin K.C. NG

Department of Surgery

The Chinese University of Hong Kong

Prince of Wales Hospital

30 - 32 Ngan Shing Street

New Territories

Hong Kong

Tel: (852) 3505 1496

Fax: (852) 3505 7974

E-mail: [kkcng95@gmail.com](mailto:kkcng95@gmail.com)

**ORCID ID** 0000-0002-3679-6368

**Twitter** @kkcng95

**Supplementary Materials - Index**

| **Supplementary Appendixes** |  |
| --- | --- |
| **Appendix S1. Patient information sheet and consent form (English Version)** | *pag. 2* |
|  |  |

**Supplementary Appendixes**

**Appendix S1.**

**Patient information sheet and consent form (English Version)**

**Asia-Pacific Multi-Center Randomized Trial of Laparoscopic versus Open Major Hepatectomy for Hepatocellular Carcinoma (AP-LAPO Trial)**

**Introduction**

You are being invited to participate in a study, which aimed to compare the immediate and long-term outcome between laparoscopic and open major hepatectomy for patients with hepatocellular carcinoma (HCC). Hepatocellular carcinoma (HCC) is the sixth most common malignancy in the world. Hepatic resection is the treatment of choice for patients with small HCC and preserved liver function. Despite low mortality rate, open hepatic resection is associated with significant morbidities (up to 30%). High incidence of tumor recurrence, and thus short survival, remain the major problems after open surgery. Laparoscopic major hepatectomy (LMH) has evolved as a minimally invasive approach. Compared with open surgery, the smaller incision in laparoscopic surgery is associated with less wound pain, less postoperative ileus, shorter hospital stay and an overall shortened recovery time. Additional theoretical benefits are the reduced immunosuppression and shorter time to initiate adjuvant medical therapies, which are particularly relevant to cancer patients. With these potential benefits, laparoscopic hepatic resection could be a better treatment option for patients with HCC than open surgery. Up till now, there is no large-scale prospective randomized trial comparing LMH with OMH for HCC in the literature.

**Research Procedure**

This is multi-center randomized trial involving 5 surgical center in Asia-Pacific regions.

If you agree to take part in this study, you will have an equal chance of being randomized in to one of the two groups, laparoscopic group or open group.

Pre-procedure investigations and assessments include routine blood tests, radiological imaging, Indocyanine green clearance test and quality of life assessment. The tumor status will be assessed by investigators to be suitable for both laparoscopic and open hepatectomy as curative measure. Both laparoscopic group and open group will involve surgery under general anesthesia. Preoperative anesthetist assessment will be carried out for all recruited patients.

*Laparoscopic group:*

Resection of the tumor will be performed under general anesthesia using laparoscopic techniques. The operating surgeon will be allowed to use various laparoscopic instruments for liver transection, including ultrasonic dissecting shears (harmonic scalpel^®^), vessel sealing system (Ligasure^®^), device using local radiofrequency energy (TissueLink^®^) and laparoscopic-adopted CUSA^®^. The specimen will be retrieved through a protected wound. The extent of resection depends on the tumor location. The aim is to obtain a 1-cm free margin in the resection.

*Open group:*

Resection of the tumor will be performed with standard open techniques as described in the principle investigator’s center. The same principle for oncological clearance is applied as in laparoscopic group.

Evaluation of postoperative outcomes

Duration of procedure, blood loss and blood transfusion related to the procedure, use of other blood products (platelet concentrate, fresh frozen plasma) will be assessed. Postoperative assessments will be evaluated. These include liver function, postoperative morbidities, operative mortality and hospital stay. No chemotherapy or any other type of adjuvant therapy will be given after treatment, as there is currently no established effective prophylactic treatment to prevent the recurrence of HCC. All patients will be followed regularly in an HCC clinic by the investigators and monitored for liver function and tumor recurrence with blood tests and imaging studies every three months. Patients with intrahepatic recurrence will be managed with laparoscopic re-resection for group A or open re-resection for group B if feasible. For patients in whom re-resection is not possible, radiofrequency ablation or trans-arterial chemoembolization will be offered as appropriate.

**Benefits**

If you participate in our study, you will not receive any monetary benefits but your participation could help us to find a better surgical approach early stage liver cancer.

**Foreseeable risks of the study**

Since this involves two different approaches of hepatectomy under general anesthesia, the foreseeable risks involve surgical risks of hepatectomy (5% mortality and 20% complication rate: bleeding and infection)

**Responsibilities of participating the study**

Extra blood samples will be collected for immunological test and the quality of life questionnaire will be offered to complete during each follow up assessment

**Privacy Confidential**

All relevant information will remain confidential and be used for research purposed only. Privacy and confidentiality will be assured throughout the study. Your name or identifying information will not be disclosed in reports or publication for this study. The personal data will be kept confidentially for ten years after the study completion. The Joint-CUHK-NTEC Research Ethics Committee is authorized to access the subjects’ records related to the study for ethics review purpose.

**Payment and extra cost**

You will not receive any payment or non-monetary sponsorship for your participation in this study. In addition, you will not be charged for any extra fee from our study.

**Voluntary**

Your participation in this study is voluntary. If you do not take part, you will not lose any benefits that you are otherwise entitled to. You may withdraw from this study at any time, and this decision will not affect your future treatment. The samples collected from the procedure may be used for the future study, you may refuse to permit your specimens to be used at some time in the future without affecting your participation in this study or your care and treatment.

**Contact**

If you have questions regarding your rights as a research subject, or if you think you have any discomforts after treatment, please call Prof. Ng Kwok Chai Kelvin who is the principal investigator for this study at 3505 1496. You can also contact the Joint CUHK-NTEC Clinical Research Ethic Committee Hotline (Tel: 3505 3935) to enquire about your rights.

**PATIENT CONSENT FORM**

**Asia-Pacific Multi-Center Randomized Trial of Laparoscopic versus Open Major Hepatectomy for Hepatocellular Carcinoma (AP-LAPO Trial)**

I am hereby to consent for my participation in this clinical trial *Asia-Pacific Multi-Center Randomized Trial of Laparoscopic versus Open Major Hepatectomy for Hepatocellular Carcinoma (AP-LAPO Trial)*.

I understand the purposes and procedures of the study and was clearly informed on possible risk that may occur during the process of this study.

I have enough time and apportunity to ask all the questions.

I understand that this study was approved by the Joint-CUHK-NTEC Clinical Research Ethics Committee.

I participate in this study on a voluntary basis.

I understand that I have the right to withdraw from this study at any time and this decision will not affect my future treatment.

Patient’s name (in block letter):____________________

Patient’s signature: ______________________________ Date: ___________ ____

Investigator (in block letter):_________________

Signature of investigator:___________________________ Date: ___________ ____

Witness’s name (in block letter):____________________

Witness’s signature:______________________________ Date: ___________ ____
